# Terapia com o Inibidor da Neprilisina e do Receptor de Angiotensina e Melhora de Parâmetros de Exercício na Insuficiência Cardíaca com Fração de Ejeção Reduzida

**DOI:** 10.36660/abc.20200566

**Published:** 2020-11-01

**Affiliations:** 1 Hospital Unimed Ribeirão Preto Ribeirão PretoSP Brasil Hospital Unimed Ribeirão Preto, Ribeirão Preto, SP - Brasil

**Keywords:** Insuficiência Cardíaca, Volume Sistólico, Fadiga, Mortalidade, Morbidade, Prognóstico, Intolerância ao Exercício

A insuficiência cardíaca com fração de ejeção reduzida (ICFER) aumentou significativamente nas últimas três décadas e está associada a alta morbidade e mortalidade.^1^ Em pacientes com insuficiência cardíaca (IC), a intolerância ao exercício sugerida pela dispneia ou fadiga aos esforços é a marca registrada da doença. Além disso, sabe-se que a qualidade de vida é acentuadamente reduzida em pacientes com ICFER. A gravidade desta limitação ao exercício e a baixa qualidade de vida têm demonstrado correlação com pior prognóstico.^2^ Para esta avaliação funcional e objetiva, o teste cardiopulmonar de exercício (TCPE) tem desempenhado um papel importante na identificação de pacientes com pior prognóstico e tem sido capaz de avaliar a eficácia de diferentes terapias para esta população com IC,^3^ como a troca de inibidores da enzima conversora de angiotensina (IECA) pelo inibidor da neprilisina e do receptor de angiotensina-neprilisina (INRA).

O estudo *Prospective Comparison of ARNI with ACEI to Determine Impact on Global Mortality and Morbidity in Heart Failure* (PARADIGM-HF) randomizou pacientes com ICFER e classe funcional da *New York Heart Association* (NYHA) II-IV para uma dose de INRA (sacubitril-valsartana) de 200 mg duas vezes ao dia ou enalapril 10 mg duas vezes ao dia, mostrando uma redução consistente de morte cardiovascular, morte por todas as causas e hospitalizações relacionadas à IC no grupo sacubitril-valsartana.^4^ Além disso, o estudo PARADIGM-HF mostrou melhora na qualidade de vida geral, conforme determinado pelo Kansas City *Cardiomyopathy Questionnaire* (KCCQ).^5^ Especificamente, as maiores limitações basais e melhoras após a administração de sacubitril-valsartana foram relacionadas a atividades como corrida e relação sexual, que podem ser um marcador substituto de melhor capacidade de esforço após a troca da terapia, ainda que muito subjetiva.

Embora as diretrizes atuais tenham endossado o TCPE como uma ferramenta ‘padrão-ouro' para avaliação prognóstica e avaliação da capacidade de esforço para pacientes com ICFER,^1^^,^^6^ faltam estudos robustos com foco em parâmetros objetivos da capacidade de exercício. Dados adicionais foram relatados em um pequeno estudo com 35 pacientes. Malfatto et al.^7^ relataram benefícios consideráveis nos parâmetros do TCPE, como aumento do consumo de pico de oxigênio (VO_2_), pulso de oxigênio e redução da inclinação VE/VCO_2_, além de melhora na fração de ejeção do ventrículo esquerdo (FEVE) e hipertensão pulmonar após seis meses de tratamento.^7^ Neste número dos Arq Brasil Cardiol, o estudo^8^ apresentou dados importantes que contribuem para o avanço do conhecimento atual de como a terapia com INRA exerce efeitos favoráveis em pacientes com ICFER.

Gonçalves et al.^8^ conduziram o estudo, um estudo aberto, não randomizado, de centro único, que incluiu 42 pacientes com ICFER (mas apenas 35 pacientes completaram o seguimento de seis meses) que apresentavam principalmente classe funcional da *New York Heart Association* (NYHA) III ou IV em 51,4% da população, e 42,9% apresentavam hospitalização prévia por IC e faziam uso de betabloqueadores (100%), IECA/BRA (100%) e antagonista do receptor mineralocorticoide (94,3%). Neste estudo prospectivo, todos os pacientes tiveram seu tratamento trocado para INRA e seguimento por 6 meses, não havendo grupo controle principalmente devido a questões éticas relacionadas à não disponibilização da terapia com INRA em pacientes com ICFER. O objetivo principal foi comparar os parâmetros do TCPE (VO_2_ pico e VO_2_ pico predito, inclinação VE/VCO_2_, limiar anaeróbio e duração do teste de esforço) antes e após 6 meses de terapia com INRAe também avaliar marcadores de remodelamento reverso através do ecocardiograma (FEVE, volume do átrio esquerdo, diâmetros diastólico e sistólico final do ventrículo esquerdo). Os pacientes foram tratados com doses crescentes de sacubitril-valsartana, com alvo de 97/103 mg duas vezes ao dia.

Neste estudo, o VO_2_ pico (14,4 ± 6,0 vs. 18,63 ± 4,9, p <0,001) e o VO_2_ pico predito (49,6% ± 18,7 vs. 65,7% ± 15,5) apresentaram um aumento importante após a introdução de INRA e também uma redução significativa da inclinação VE/VCO_2_ (36,7 ± 7,2 vs. 31,1 ± 5,8, p <0,001). Essas variáveis do TCPE têm sido fortemente relacionadas ao prognóstico da IC e sua melhora está bem correlacionada a melhores desfechos.^9^ Além disso, os benefícios dos parâmetros do TCPE também foram observados nos subgrupos com dose alvo de INRA submáxima.

Além disso, houve um aumento na duração do exercício durante o teste cardiopulmonar e isso provavelmente reflete o benefício relatado na classe da NYHA após o tratamento, o qual foi relatado como uma porcentagem significativa (74,3%) de pacientes que apresentaram ao menos melhora de uma classe da NYHA.

Essas mudanças favoráveis de classe funcional da NYHA também foram relatadas em uma coorte retrospectiva do mundo real, embora com uma porcentagem menor de pacientes que melhoraram a classe da NYHA. Lau et al.^10^ coletaram dados basais e de seguimento em 201 pacientes que receberam sacubitril-valsartana e foram acompanhados por 221 ± 114 dias.^10^ Em contraste, os dados do TCPE do mundo real não mostraram uma diferença significativa em 45 pacientes recebendo INRA, mas houve uma melhora de atividade do paciente em domicílio. Esses achados divergentes podem ser explicados pelo fato de que a população do mundo real era mais velha e menos sintomática (mais pacientes em classe II da NYHA, o que pode reduzir o efeito da terapia), junto com as limitações de uma coorte observacional retrospectiva e coleta incompleta de dados.

Avaliando a capacidade do tratamento com INRA em promover remodelamento reverso através de parâmetros ecocardiográficos, o estudo também mostrou aumento significativo da FEVE média, de 5,9%, com evidências de remodelamento reverso (menor volume do ventrículo esquerdo e também do átrio, redução da pressão sistólica arterial pulmonar). A melhora desses parâmetros ecocardiográficos foi muito semelhante à magnitude do remodelamento reverso observada no estudo PROVE-HF, que mostrou um aumento na média da FEVE em seis meses (5,2%) e doze meses (9,4%).^11^

Existem possíveis implicações clínicas destes 2 estudos (PROVE-HF e de Gonçalves et al.^8^). Em primeiro lugar, é provável que o remodelamento reverso observado promova uma melhora significativa na FEVE e possa evitar a terapia com cardioversor/desfibrilador para prevenção primária. Além disso, os benefícios impressionantes para o TCPE e a NYHA deste estudo provavelmente serão traduzidos em uma melhor qualidade de vida e capacidade funcional, podendo também reduzir a indicação de terapia de ressincronização cardíaca para alguns pacientes com IC. Finalmente, o estudo Gonçalves et al.,^8^ acrescenta evidências favoráveis às recomendações baseadas em evidências sobre a eficácia do INRA e fornece outras iniciativas para a ampla disseminação desse tratamento para pacientes com ICFER.^12^

Entretanto, o estudo de Gonçalves et al.,^8^ assim como o estudo PROVE-HF, não foi randomizado, e a ausência de um grupo controle representa uma limitação considerável, além do fato de que os efeitos notáveis nos parâmetros do TCPE podem estar relacionados a outras terapias para a IC. Além disso, os pacientes com IC do estudo de Gonçalves et al.,^8^ eram mais graves do que os pacientes dos estudos PARADIGM-HF e PROVE-HF, com uma porcentagem maior de pacientes com hospitalização prévia relacionada à IC e mais pacientes com NYHA III e IV, o que provavelmente influenciou na magnitude do benefício da terapia com INRA e pode, em parte, explicar a falta de benefício no TCPE em uma coorte de pacientes do mundo real.^10^ Com relação às variáveis do TCPE, alguns parâmetros importantes estavam ausentes, como ventilação oscilatória durante o esforço, trajetória do pulso de oxigênio, inclinação da eficiência do consumo de oxigênio e pico do PETCO_2_, os quais poderiam ter acrescentado informações sobre o efeito do INRA nessa população.

Em conclusão, o estudo de Gonçalves et al.,^8^ publicado nesta edição dos Arq Brasil Cardiol, sugere fortemente que a terapia com INRA pode promover mudanças significativas na capacidade funcional e nos parâmetros obtidos pelo TCPE, bem como uma melhora considerável nas variáveis ecocardiográficas relacionadas ao remodelamento reverso de pacientes com ICFER. Embora este não seja um ensaio randomizado, certamente adiciona mais dados benéficos da terapia com INRA na população com ICFER. Especificamente para a morbidade relacionada à IC, abordagens mais direcionadas são necessárias para fornecer uma melhor qualidade de vida e melhores desfechos clínicos.
