# Supplementary material for: Terapia com o Inibidor da Neprilisina e do Receptor de Angiotensina e Melhora de Parâmetros de Exercício na Insuficiência Cardíaca com Fração de Ejeção Reduzida
Source: Arq Bras Cardiol. 2020 Nov 1;115(5):828–9. [Article in Portuguese] doi: 10.36660/abc.20200566 (PMC8452205; doi:10.36660/abc.20200566)
Supplement: Translation

# Translation: Angiotensin Receptor-Neprilysin Inhibition Therapy and Improved Exercise Parameters in Heart Failure with Reduced Ejection Fraction

**Affiliations:** 1 Ribeirão PretoSP Brazil Hospital Unimed Ribeirão Preto, Ribeirão Preto, SP - Brazil

**Keywords:** Heart Failure, Stroke Volume, Fatigue, Mortality, Morbidity, Prognosis, Exercise, Intolerance

Heart failure (HF) with reduced ejection fraction (HFrEF) has increased significantly in the last three decades and is associated with high morbidity and mortality.^1^ In patients with HF, exercise intolerance suggested by dyspnea or fatigue during exertion is the hallmark of the disease. Additionally, health-related quality of life is known to be markedly reduced in HFrEF patients. The severity of this exercise limitation and low quality of life has been shown to correlate to worse prognosis.^2^ For this functional and objective assessment, cardiopulmonary exercise testing (CPET) has played an important role on identifying those worse-prognosis patients and has been able to evaluate the effectiveness of different therapies for this HF population,^3^ such as the switch of angiotensin-converting enzyme inhibitors (ACEI) to angiotensin receptor neprilysin inhibition (ARNI).

The Prospective Comparison of ARNI with ACEI to Determine Impact on Global Mortality and Morbidity in Heart Failure (PARADIGM-HF) trial randomized HFrEF patients with New York Heart Association functional class (NYHA) II-IV to the angiotensin receptor neprilysin inhibitor (sacubitril-valsartan) 200 mg twice daily or enalapril 10 mg twice daily and showed a consistent reduction on cardiovascular death, all-cause death and HF-related hospitalizations in the sacubitril-valsartan group.^4^ Moreover, the PARADIGM-HF showed improvement in overall quality of life as determined by the Kansas City Cardiomyopathy Questionnaire (KCCQ).^5^ Specifically, the greatest baseline limitations and improvements after sacubitril-valsartan were related to activities such as jogging and sexual intercourse, which might be a surrogate marker of better exercise capacity after switching the therapy, although very subjective.

Even though that current guidelines have endorsed the CPET as a gold-standard tool for prognostic assessment and exercise capacity evaluation for HFrEF patients,^1^^,^^6^ larger trials focusing on objective parameters on exercise capacity are lacking. Additional data has been reported in a small study with 35 patients. Malfatto et al.^7^ reported considerable benefits on CPET parameters such as the increase in peak oxygen consumption (VO_2_), oxygen pulse and the reduction in V_E_/VCO_2_ slope, along with the improvement on left ventricular ejection fraction (LVEF) and pulmonary hypertension after six months of treatment.^7^ In this issue of *Arquivos Brasileiros de Cardiologia*, the study^8^ presented important data that contributes to the advance of current knowledge of how ARNI therapy exerts favorable effects in patients with HFrEF.

Gonçalves et al.^8^ conducted the study, an open-label, non-randomized, single-center investigation that included 42 HFrEF patients (but only 35 patients completed the six-month follow-up) who primarily had NYHA class III or more in 51,4% of the population and 42,9% had had a previous HF hospitalization and were taking beta-blockers (100%), ACEI/ARB (100%) and mineralocorticoid receptor antagonist (94,3%). In this prospective study, all patients have been switched to ARNI and followed by 6 months and there was no control group mainly related to ethical concerns of withholding ARNI therapy in HFrEF patients. The main objective was to compare CPET parameters (peak VO_2_ and peak predicted VO_2_, V_E_/VCO_2_ slope, anaerobic threshold, and duration of the exercise test) before and after 6 months of ARNI therapy and also evaluated markers of reverse remodeling through the echocardiogram (LVEF, left atrium volume, left ventricle end-diastolic and end-systolic diameters). Patients were treated with escalating doses of sacubitril-valsartan, targeting 97/103 mg twice daily.

In this study, peak VO_2_ (14.4 ± 6.0 vs 18.63 ±4.9, p<0.001) and peak predicted VO_2_ (49.6% ± 18.7 vs 65.7% ± 15.5) presented an important increase after the introduction of ARNI therapy and also a significant reduction in V_E_/VCO_2_ slope (36.7 ± 7.2 vs 31.1 ± 5.8, p<0.001). Those CPET variables have been strongly related to HF prognosis and their improvement is well correlated to better outcomes.^9^ Additionally, the CPET parameters benefits were also observed in the non-maximal target ARNI dose subgroups.

Furthermore, there was an increase in the duration of exercise during the test and it is likely reflecting the reported benefit on the NYHA class after the treatment, which was reported as an impressive percentage (74,3%) of the patients that presented at least one NYHA class improvement.

Those favorable NYHA class changes were also reported in a real-world retrospective cohort although with a lower percentage of the patients that improved the NYHA class. Lau et al.^10^ collected baseline and follow-up data in 201 patients that received sacubitril-valsartan and were followed by 221 ± 114 days.^10^ In contrast, the real-world CPET data have not shown a significant difference in 45 patients under ARNI therapy but there was an improvement in the patient-level activity at home. Those divergent findings could be explained by the fact that the real-world population was older and less symptomatic (more NYHA class II which might reduce the effect of the therapy), along with the limitations of a retrospective observational cohort and incomplete data collection.

Assessing the ability of the ARNI treatment to promote reverse remodeling through echocardiographic parameters, the study also showed a significant increase in the mean LVEF of 5.9% and there was evidence of reverse remodeling (lower left ventricular volumes as well as atrium volumes, reduction on pulmonary artery systolic pressure). These echocardiographic parameters improvements were very similar to the magnitude of the reverse remodeling observed in the PROVE-HF trial, which showed an increase in the mean LVEF at six months (5.2%) and twelve months (9.4%).^11^

There are clinical implications from these 2 studies (PROVE-HF and by Gonçalves et al.^8^). First of all, the observed reverse remodeling is likely to promote a significant improvement in LVEF and might avoid cardioverter-defibrillator therapy for primary prevention. Additionally, the impressive CPET and NYHA benefits of this current study are likely to be translated into a better quality of life and functional capacity and might also preclude the cardiac-resynchronization therapy indication for some HF patients. Last, Gonçalves et al.^8^ study adds supportive evidence to the evidence-based recommendations of ARNI efficacy and provide further initiatives to the widespread dissemination of this treatment to HFrEF patients.^12^

Nevertheless, the Gonçalves et al.,^8^ study, as well as the PROVE-HF trial, were not randomized trials and the absence of a control group represents a considerable limitation and the notable effects on the CPET parameters might be related to other HF therapies. Besides, the HF population of the by Gonçalves et al.,^8^ study was sicker than PARADIGM-HF and PROVE-HF trials, with a higher percentage of patients with a previous HF-related hospitalization and more patients with NYHA III and IV, which is likely to have influenced the magnitude of the benefit of ARNI therapy and might in part explain the lack of CPET benefit in a real-world cohort of patients.^9^ Regarding CPET variables, there were some important missing parameters such as exercise oscillatory breathing ventilation, oxygen pulse trajectory, oxygen uptake efficiency slope, and peak PETCO2 that could have added insights into the effect of ARNI in that population.

In conclusion, the Gonçalves et al.,^8^ study reported in this issue of *Arquivos Brasileiros de Cardiologia* strongly suggests that ARNI therapy can promote significant changes in the functional capacity and measured CPET parameters of exercise tolerance as well as a considerable improvement on echocardiographic variables related to reverse remodeling of HFrEF patients. Although this was a not randomized trial, it certainly adds more beneficial data of the ARNI therapy in the HFrEF population. Specifically for the HF-related morbidity burden that characterizes this disease, more targeted approaches are warranted to provide a better quality of life and health-related outcomes.
